# The relationship between intolerance of uncertainty in chiropractic students and their treatment intervention choices

**DOI:** 10.1186/s12998-017-0150-2

**Published:** 2017-07-19

**Authors:** Stanley I Innes, Charlotte Leboeuf-Yde, Bruce F Walker

**Affiliations:** 1 School of Health Professions, Murdoch University, Murdoch, Australia; 2 Institut Franco-Européen de Chiropraxie, Ivry sur Seine, Paris, France; 3 CIAMS, Univ. Paris-Sud, Université Paris-Saclay, 91405 Orsay Cedex, France; 4 CIAMS, Université d’Orléans, 45067 Orléans, France

**Keywords:** Intolerance of uncertainty, Practice patterns, Chiropractic, Education

## Abstract

**Background:**

Psychological factors, such as intolerance of uncertainty (IU), have been shown to impact on the quality of medical care. However, this psychological measure has not been studied in the chiropractic profession. Our objective was to investigate if higher levels of IU in chiropractic students were related to poor choices of management in specific clinical scenarios. Also, we sought to investigate if levels of IU were related to students’ intentions to adopt a prescriptive chiropractic technique system and evaluate their levels of self-belief.

**Method:**

Between October and November of 2016, students from two Australian chiropractic programs (*N* = 444) answered a questionnaire on measures of IU levels, patient case scenarios for neck and low back pain, and questions about self-ratings of their future chiropractic abilities and perceived need for the adoption of a chiropractic technique system. Associations were tested by the IU score and the therapeutic choices relating to a) a neck pain case scenario, b) a low back pain scenario, c) various technique systems, and d) the self-rated competence level treating the IU score both as a continuous and a categorical variable.

**Results:**

There was an overall response rate of 53%. Those students who were high in levels of IU were significantly more likely to make incorrect clinical decisions than those with normal or low levels of IU for the neck pain case scenario. No differences were found on the low back pain scenario, on preferences to use a technique system in the future, or on predicted self-rating of competence after graduation.

**Conclusions:**

Psychological factors, such as IU, may have an impact on chiropractic students’ clinical decisions. However, it does not impact on all aspects of practice. This finding has implications for chiropractic educators, especially when dealing with neck pain. However, it may be relevant to continue the search for specific personality profiles in relation to various favourable and unfavourable practice patterns, as it is unknown whether these dynamics are important for other aspects of chiropractic education.

**Electronic supplementary material:**

The online version of this article (doi:10.1186/s12998-017-0150-2) contains supplementary material, which is available to authorized users.

## Background

Professions accepted into the mainstream health care system are expected to provide high quality care. In chiropractic, the educational institutions have a responsibility to select and educate students to this end. Failure in this task may have undesirable consequences for chiropractors, patients, and public health.

External circumstances, such as educational facilities, curriculum, and staff are not solely responsible for graduate attributes. Intrinsic factors within students will also influence educational outcomes and ultimately practice standards. Clearly, psychological profiles play an important role in determining human behaviour, both positively and negatively. In this context, intolerance of uncertainty (IU) could be one such psychological factor. IU refers to a dispositional characteristic that reflects a set of negative beliefs about uncertainty and its implications and represents an underlying fear of the unknown [1, 2]. IU has also been described as an incapacity to endure the aversive response triggered by the absence of salient, key or sufficient information [3]. These negative beliefs about uncertainty in everyday situations are inflexible and result in a desire for predictability [4, 5]. Consequently, IU has been found to be associated with behavioural performance [6]. With such an influence on behaviours, it is not surprising that past research has shown this factor to impact on the decision-making of primary care medical / general practitioners [7].

Specifically, higher anxiety shown as high levels of IU is associated with lower compliance with evidence-based guidelines, manifesting in higher frequency of ordering diagnostic tests, more variability in treatment options for an individual [8], and generally increased resource use in the health care system by medical / general practitioners [7]. It has been estimated that physicians’ mean medical management costs increased by 17% for each standard deviation that uncertainty scores increased [7]. In the chiropractic profession, the use of maintenance care in patients who do not improve with treatment could be seen as an example of increased use of resources. This example has been demonstrated in three previous surveys of chiropractors’ choice of treatment strategies in patients with low back pain [9–11].

IU has been shown to be associated with lower levels of confidence with decision making, a reduced likelihood to change any previously made decisions despite receiving new information, an increased likelihood to seek additional information (e.g. clinical tests) when not required, and a propensity to react and behave overly cautiously even in low levels of perceived threat [2, 12]. However, the art of clinical practice involves learning to deal with varying levels of uncertainty.

IU is also strongly related to anxiety and this can influence thoughts about one self [13, 14]. Anxious people are more likely to have negative internal working models of themselves [15, 16]. This manifests in the belief that they may not have the resources to cope with uncertain events and are thus more likely to defer to an external source for answers [17]. However, past research has also found that high levels of confidence may result in lower levels of accuracy in clinical diagnosis in medical students and physicians [18]. This would also suggest that IU may be important in clinical decision making.

In contrast, those who have low levels of IU have been shown to be more psychologically flexible, less distressed by uncertainty, and enjoy higher levels of psychological health [2, 19]. Further they are more likely to act in a timely and measured manner [20].

There is a rich history of research exploring the relationship between IU and the behaviour of health care practitioners. However, nothing is known about how IU impacts on chiropractors’ self-esteem and clinical practice. For example, would chiropractors who are intolerant of uncertainty be more comfortable with prescriptive technique systems that indicate ‘where the problem is’ and ‘how to treat it’, rather than accepting the grey shades of clinical reality?

In chiropractic technique systems, stereotyped answers to ‘where the problem is’ could allow a purely technical approach such as a Derifield Test for leg-length inequality to identify a biomechanical problem in the pelvis [21], an x-ray analysis for a ‘subluxated’ vertebra [22], sacro-occipital pelvic blocking based on body sway categorisation [23], and an ‘Applied Kinesiology therapy localisation’ for area of problem [24]. Examples of ‘how to treat’ in chiropractic technique systems could be a recipe-based approach, such as the Gonstead x-ray analysis, as it provides information on the side of adjustment and line of drive [25], or an Applied Kinesiology ‘challenge’ for direction of thrust [24].

The alternative to a purely technical approach is a flexible and open one based on a multitude of findings assimilated without the need for pre-set rules. The first approach is formulaic and tends to reduce the role of the chiropractor to becoming ‘technician-like’, while the second approach fits in with the societal and regulatory expectations of a licenced qualified health care professional [26].

Studies have identified various profiles of chiropractic practice, some of which were labelled unsuitable [27, 28]. These practice styles are typified by traditional chiropractic ‘philosophical’ beliefs, technique styles that make recommendations partially or wholly incompatible with evidence-based care, and excessive X-ray usage [28]. A research question in this context is: “Do higher levels of IU play a role in chiropractors choosing to adopt out-dated treatment systems?” This question is important, as such a profile, in turn, may result in unsuitable practice behaviour.

In people with high anxiety scores, it has been established that emotion-laden choices with low probabilities are overweighted and high probabilities are underweighted [29–31]. Thus, when presented with a low threat clinical scenario with multiple options, anxious medical / general practitioners (such as those with higher IU) tend to prematurely seek unnecessary clinical tests or inappropriately refer to another practitioner [7]. In chiropractic, this could perhaps result in a dichotomy of both an overly careful attitude and an unsuitable careless approach. In the first case, chiropractors belonging to this category are likely to refer their patients for inadequately founded reasons or seek second opinions in slightly complicated cases. Alternatively, when presented with a high threat clinical scenario, practitioners with high levels of IU are likely to display the tendency to downplay the risk and not order the appropriate clinical tests or fail to refer for more appropriate care.

Previous studies using specific case scenarios have shown that chiropractors in various countries do not all appear to choose logical treatment strategies for their patients [9–11]. This may be related to intrinsic factors, such as a higher IU.

The intention of this study was to investigate if higher IU was related to poor choices of chiropractic management in specific clinical scenarios. Specifically, our objectives were to answer the following questions:

1. Is there a difference between IU groups (high versus normal or low levels) in their approach to:

- a neck pain scenario?
- a low back pain scenario?

2. Is there a difference between these IU groups in their attitudes to the use of recipe-like technique systems?
3. Is there a difference between IU groups’ self-rating of their own skills?

## Methods

### Study procedure

A team consisting of the three authors designed the questionnaire and four 4th year students from Murdoch University assisted with the survey administration and data collection.

This cross-sectional study was conducted between October and November in 2016. The chiropractic programs based at two Australian universities (Murdoch University and Macquarie University) were used for data collection. This was a quantitative descriptive study using an anonymous classroom handout questionnaire, as this approach facilitated the collection of a large amount of robust data in a timely and cost-effective manner. The entire student population across both programs was invited to participate and consent was obtained prior to completion of the survey.

Ethics approval was granted by Murdoch University (Project No 2016/118) and was classed as negligible risk research.

The project followed the same protocols in both institutions, consent was obtained from students, data were non-identifiable (anonymous) and permission was obtained from the Head of the Macquarie University chiropractic program to conduct the research. Accordingly, the study met the criteria for classification under the National Statement on Ethical Conduct of Human Research (2007) (Sections 5.1.8 and 5.1.22) as exempt from requiring ethics approval from both Universities.

### The questionnaire

The survey contained four sections (see Additional file 1), with the results of some sections to be reported elsewhere. The first section sought demographic details (chiropractic program, sex, year of study) as studies on medical students indicate that levels of IU decrease over the course of training [32]. Further, age and sex have been shown to be independent predictors of IU [33].

In the second section, we devised direct questions to determine attitudes towards chiropractic care and clinical behaviour. The survey also sought students’ attitudes toward their likelihood of giving advice in their own chiropractic clinics and their beliefs on the capacity of spinal manipulation to impact on a number of health issues. It also asked students about their beliefs on the need, once graduated, to adopt a rigid comprehensive chiropractic technique system, which would inform them of the patient’s presenting problem (i.e. a technique system used to reduce uncertainty).

The third section was comprised of two clinical scenarios from previously published case management scenarios with chiropractors [9, 34]. A full description of the rationale for each clinical scenario and the rationale for classification of correct and incorrect choices is attached (see Additional file 2).

In the first case study, five scenarios were presented, beginning with a simple uncomplicated case of neck pain, which gradually progressed through to a scenario requiring immediate medical referral. The neck case consisted of the following general information: “A 28-year old man, tennis player by profession, consults you for a right-sided intense neck pain without any radiating pain. You note an antalgic position of the head, no other musculoskeletal signs (eg., no acute torticollis), no other health problems in particular, normal x-rays for his age, and no signs of alert (red flags).” [34] There was a choice of six answers for each of the five scenarios ranging from treating the patient on his/her own through to not providing treatment and arranging referral.

In a previous study, consensus was demonstrated on the most appropriate management choices across the five scenarios, thus allowing differentiation between chiropractors who select appropriate and inappropriate intervention strategies [34]. The inappropriate choices related to referral out of the chiropractic clinic when the patient should have been treated by the chiropractor and also to continued care when the patient should have been referred out.

The second case described a range of clinical scenarios for a patient with low back pain designed to identify which management strategies the chiropractors preferred to use in response to various outcomes of the initial treatment program [9]. This questionnaire had nine possible outcome scenarios that were briefly described. Six clinical management alternatives were offered for each outcome scenario. The basic facts for this hypothetical patient were: “A 40-year old man consults you for low back pain with no additional spinal or musculoskeletal problems, and with no other health problems. His X-rays are normal for his age. There are no ‘red flags’”.

The nine additional scenarios were constructed in such a way as to include cases that went from uncomplicated to more difficult, including scenarios with no past history of low back pain, those with intermittent low back pain over the past year, and those with several similar events over the past year. In three previous studies, a pattern of self-reported clinical management strategies was demonstrated which allowed identification of those clinicians who did and did not follow ‘clinically logical’ answers (see Additional file 2) [9–11].

The fourth section consisted of the Intolerance of Uncertainty Scale. We chose the validated 12-question version (IUS-12) that utilises a 5-point Likert scale with responses ranging from ‘not at all characteristic of me’ to ‘entirely characteristic of me’ [1, 35–38]. Examples of questions included ‘unforeseen events upset me greatly’ and ‘the smallest doubt can stop me from acting’. The maximum possible score was 60, reflecting high levels of intolerance of uncertainty.

Lastly, we asked students to rate themselves as a chiropractor compared with other chiropractic students in their class.

### Procedure

The content and wording of the questionnaire were pilot-tested by a small number of chiropractors. The questionnaire was adjusted in response to their comments and tested on a small number of chiropractic students. This process detected some logical errors in the description of the attitudes and beliefs and resulted in some wording changes, which further improved the content, wording and design of the questionnaire.

Students in both chiropractic programs were informed that participation was voluntary and would be anonymous. Students in years 1 and 2 were not given the case scenarios in their survey, as they were deemed to have inadequate clinical knowledge.

### Variables of interest and analysis of data

Data were entered and analysed in SPSS v.22 (IBM Corp, Armonk NY, USA) after identifying and correcting any incomplete or corrupt data. All survey items were dummy variable coded and descriptive statistics generated.

#### Predictor variable

Visual examination of the distribution of the IU scores suggested a cut-off score at scores of 36 and above for construction of the two IU groups. Receiver operator characteristic (ROC) curves were utilised to evaluate potential high IUS-12 from total IUS-12 score. Sensitivity and specificity of the coordinate points of the resulting ROC curve were used to identify the potential cut-off score for IUS-12. The optimum critical value for the IUS-12 was identified as 35.5. Subsequently scores of 36 and above were allocated into the High-IU group (25% of the students) and those below this score were allocated into a Normal-to-low IU group (75% of the students). This decision was also supported by previous published research with non-clinical samples which used similar values [39, 40].

#### Outcome variables

##### Variable 1: Neck Pain Case

From the previously published neck pain case [34], the research team determined which answers were the correct and incorrect treatment choices for each of these five scenarios. These are further explained in Additional file 2.

Students were given one point for selecting the incorrect treatment choice for the first two and last two scenarios, making it possible to obtain a score of between zero and four. For items 1 and 2, all the answers other than treating the patient on his own (response A) were considered incorrect. For items 4 and 5, all the answers other than choosing not to treat the patient but instead, referring the patient out (response E), were considered incorrect.

##### Variable 2: Low Back Pain Case

A previously published low back pain case was used, on the basis of which students were asked to identify their clinical decisions [9]. The research team determined which answers were correct and incorrect for i) inappropriate selection of premature referral (items 1,2,4; scores ranging from 0 to 3) and ii) inappropriate selection of continuation of care when referral was indicated (items 6,7,8,9; scores ranging from 0 to 4). Thus, high scores were indicative of an inappropriate management choice. These questions and their rationale for the ‘correct’ and ‘incorrect’ answers are described in Additional file 2.

##### Variable 3: Use of chiropractic technique analysis / evaluation systems

The answers to the question on chiropractic technique were dichotomised into yes (‘yes’ and ‘yes probably’) and no (‘don’t know’, ‘no, probably not’, ‘no’).

##### Variable 4: Belief in Self

The rating of students’ own future capacity as a chiropractor was grouped into four categories: Below Average (‘below average’ and ‘a bit below average’), Average, Above Average (‘A bit above average’ and ‘above average’), and Don’t Know. Preliminary analysis revealed the Below Average groups to be very small, and accordingly, we combined these respondents with the Don’t Know group, as we considered both types to indicate uncertainty.

Initially, the mean IU was compared for the chiropractic programs, sex, and year of program to see if subgroup analyses would be necessary. For this, tests for significant differences and relationships were carried out with Chi-square, ANOVA and linear regression analyses depending on the type of variable and data distribution.

The associations between the IUS-12 score and the scores for the neck pain and low back pain scenarios were tested in two ways. The score was treated as a continuous variable in a linear regression analysis, as this would make most use of the data. In addition the IUS-12 score was dichotomized into high and normal-to-low IU groups and tabulated with the numbers of correct and incorrect scores on the neck and low back case scenarios, as this would allow for a clinical interpretation of the results. We expected that the two types of analyses would go in the same direction.

Thereafter, associations between Normal-to-low and High IU and the predictor variables were tested for significance using the Chi-square test. Estimates were reported with 95% confidence intervals (CI). Preliminary analyses revealed no links between these variables. For this reason and because of small numbers, no sub analyses by institution, sex, or year of program were carried out in relation to tests of association.

To determine if there was a difference between IU groups in the students’ approaches to the neck pain case and also to the low back pain case (on both the aspect of maintenance care and inappropriate referrals), responses were dichotomised using the threshold that zero incorrect answers were treated as ‘acceptable’ answers and one or more incorrect replies were treated as ‘unacceptable’.

## Results

### Descriptive data

Of a possible 313 Murdoch University chiropractic students, 216 (69%) completed the survey and of a possible 518 Macquarie University chiropractic students, 228 (44%) completed the survey, giving a total of 444 students, of whom 224 were male (50%). This sample of 444 from a sampling frame of 831 possible participants gave an overall response rate of 53%. As can be seen in Table 1, there was no difference in IUS-12 scores between institutions or between sexes, whether tested by year of program or for the whole institutional sample. Consequently, the two programs were combined for all subsequent analyses. The mean IUS-12 score (maximum possible score = 60) for the Normal-to-low IUS-12 group was 26.5 (SD = 5.6) and for the High IUS-12 group was 40.2 (SD = 4.2).

**Table 1:** School, sex, year of program, and students’ mean Intolerance of Uncertainty score in a study of Australian chiropractic students

Year of Program	Males/Females	% of respondents’ year	IU score Males (0 to 60)(SD)	IU score Females (0 to 60)(SD)
1st year MQ	43/34	^c^	34.2 (8.0)	31.7 (7.7)
MU	31/45	62%	30.9 (6.7)	30.5 (7.7)
2nd year MQ	17/10	^c^	33.9 (8.2)	26.8 (9.6)
MU	17/33	46%	34.1 (6.2)	32.6 (8.5)
3rd year MQ	42/20	^c^	31.1 (7.8)	31.2 (8.5)
MU	19/22	62%	28.3 (10.1)	27.6 (8.3)
4th year MQ	34/25	^c^	28.4 (8.5)	27.6 (8.1)
MU	6/21	79%	27.8 (9.7)	26.1 (6.4)
5th year MQ	3/0	^c^	29.3 (8.6)	
MU	12/10	55%	28.0 (9.7)	22.3 (3.6)
All Years MQ	139/89	69%	31.7 (8.3)	29.2 (8.1)
MU	85/131	44%	30.2 (8.2)	29.9 (8.3)

^c^denotes information not available because students were enrolled across differing years. IUS-12 Cronbach’s alpha was 0.87 (skewness 0.20 (SD 0.11), Kurtosis −0.59 (SD 0.23)0

All variables of interest have been described in Table 2. As can be seen, approximately 70% were classified as having a ‘normal’ IU, using our arbitrary cut point. The most common answers for each outcome variable are described below. Almost 80% would have made at least one inappropriate referral for the neck case scenario, whereas about 80% would not have made an inappropriate referral in the low back pain scenario and almost 70% would not have inappropriately recommended maintenance care. Similarly, approximately 80% were positively inclined towards the use of a technique system of analysis. Just over 50% self-rated their predicted clinical competence as above average and only 3% as below average.

**Table 2:** Variables of interest in a study of Australian chiropractic students

Variables of interest		All N (%)
IU	Normal	307 (69)
	High	126 (28)
	Missing	11 (3)
^a^Neck Pain Scenario: Number of inappropriate referrals		
O		44 (21)
1		93 (44)
2		48 (23)
3		17 (8)
4		9 (4)
Missing		2(1)
^a^Low back Pain Scenario: Number of inappropriate choices of maintenance		
0		139 (68)
1		43 (21)
2		12 (6)
3		3 (1)
4		5 (2)
Missing		3 (1)
^a^Low Back Pain Scenario: Number of inappropriate referrals		
0		177 (83)
1		22 (10)
2		3 (1)
3		2 (1)
Missing		9 (4)
Preference for Technique System of Analysis		
Yes		209 (47)
Yes, probably		156 (35)
Don’t know		55 (12)
o, probably not		18 (4)
No		5 (1)
Missing		0
Self-rating of predicted clinical competence		
Below Average		11 (3)
Average		109 (25)
Above Average		240 (54)
Don’t know		74 (17)
Missing		10 (2)

^a^These questions were submitted only to students in years 3, 4 and 5

### Was there a difference between IU groups in the students’ approaches to a neck pain case and a low back pain case?

High IU groups were significantly more likely than Normal-to-low IU groups to make incorrect clinical decisions about the neck pain case. No significant differences were found comparing High and Normal-to-low IU groups’ numbers of inappropriate choices of maintenance care in the low back pain case. No significant differences were found comparing High and Normal-to-low IU groups’ number of inappropriate referrals in the low back pain case (Table 3).

**Table 3:** Associations between Intolerance of Uncertainty and various clinical outcome variables in a study of Australian chiropractic students

Clinical outcome variables	Numbers of students with Normal IU N (%)	Numbers of students with High IU N (%)
^a^Neck Pain Scenario: Number of inappropriate referrals (*N* = 206)		
Acceptable	113 (71)	22 (48)
Unacceptable	47 (29)	24 (52)
χ^2^ (1, *N* = 206) = 8.2, *p* = .004		
^a^Low Back Pain Scenario: Number of inappropriate choices of maintenance care (*N* = 198)		
Acceptable	109 (71)	29 (64)
Unacceptable	44 (29)	16 (36)
χ^2^ (1, *N* = 198) = .76, *p* = .38		
^a^Low Back Pain Scenario: Number of inappropriate referrals (*N* = 200)		
Acceptable	136 (88)	37 (82)
Unacceptable	19 (12	8 (18)
χ^2^ (1, *N* = 200) = .91, *p* = .34		
Preference for Technique System of Analysis (*N* = 432):		
Yes	247 (81)	108 (86)
Don’t know/ No	60 (20)	17 (14)
χ^2^ (1, *N* = 432) = 2.14, *p* = .14		
Self-rating of predicted clinical competence (*N* = 432)		
Below Average	7 (2)	4 (3)
Average	76 (25)	32 (25)
Above Average	177 (58)	63 (50)
Don’t know	46 (15)	27 (21)
χ2 (3, N= 432) = 3.4, p = .33		

^a^these questions were only submitted to students in years 3, 4 and 5

These results were confirmed with the linear regression analysis that was used to test if IUS-12 scores significantly predicted participants’ scores on the neck and low back pain case scenarios and their future self-rating as chiropractors. The results of the regression indicated that IUS-12 significantly predicted the response of the scores on the neck pain scenario (F(1200) = 12.46, *p* = .001, R^2^ = 0.05). However, IUS-12 scores were not found to be a significant predictor for scores on the low back pain case scenario (F(1188) = 1.85, *p* = 0.18. R^2^ = .004) or for future self-rating scores (F(1430) = 0.55, *p* = 0.46, R^2^ = .001).

### Was there a difference between IU groups in their attitudes to use of ‘recipe-like’ technique systems?

No significant difference was found comparing High versus Normal-to-low IU groups’ preferences for wanting to use a technique system of analysis.

### Was there a difference between IU groups in their attitudes on self-rating of own skills?

No significant difference was found comparing High versus Normal-to-low IU groups’ preference or self-rating of their predicted clinical competence after graduation. When the highest and lowest self-esteem groups were compared, the percentage estimates indicated that those high in IU were more likely to rate themselves as ‘below average’ or as ‘don’t know’ (highest self-esteem group = 33%, lowest self-esteem group = 23%) and less likely to rate themselves as ‘above average’ (67% and 77% respectively). However these findings were non-significant (*p* = 0.6).

## Discussion

### Summary of findings

To the best of our knowledge, this is the first study to investigate personality traits in chiropractic students and the impact on their clinical decision-making. The neck pain scenario was clearly a challenge to the students, whereas they appeared more knowledgeable on the low back pain scenario, judging by the fewer numbers of inappropriate choices. The vast majority were positive towards the use of a prescriptive technique system but, interestingly, almost no one rated themselves as below average in predicted clinical confidence.

A High IU predicted an unacceptable approach to the neck pain scenario, either by referring out too early or not referring out where necessary. None of the other outcome variables were significantly associated with the IU.

### Discussion of findings and comparison with other studies

This chiropractic student population had IU scores that were similar to previous Australian university studies involving medical and psychology students [41, 42]. Also, their IU scores were sex-invariant as has been previously shown in other cohorts [37]. The only outcome variable to be sensitive to the IU was the neck pain case. High IU was indeed associated with unsuitable choices. Perhaps this can be explained by the nature and structure of the cases. The neck pain case clearly being more alarming than the low back pain case could have made it more anxiety-provoking, particularly in students who do not cope well with uncertainty. The potential for serious consequences from cervical spine manipulation, such as stroke from a cervical artery dissection, is a likely contributor to creating increased anxiety levels, when considering manipulative treatment of the neck.

The reason for the ‘non-finding’ of the low back pain vignette and IU was unclear. Clearly there is the potential for adverse outcomes, although uncommon, with chiropractic care also for lower back pain. The difficulty and complexity of identifying the pain generator in low back pain is well documented [43] but perhaps students are not aware of this, thus feeling much more at ease with low back pain than with neck pain. Therefore, anxiety could be perhaps be more manifest in practicing chiropractors with high levels of IU and this warrants further investigation.

More recent research has suggested that measures of IU should be designed around the specific situations in which they occur [1, 2, 44]. For example the items in the IUS-12 could be altered in future studies to make them specific to the diagnosis and treatment of lower back pain to improve our capacity to understand the role of IU in low back pain care.

On the finding that the desire to acquire a stereotypical technique system was not associated with high IU, a number of reasons for this result are possible. One hypothesis is that students do not realise that such techniques are based on largely ‘belief-based’ rules and also do not understand that the body’s so-called innate response to human dis-ease and disease cannot be simplified in such a manner, as is often purported in these technique systems.

It is common that people over-rate their own abilities [45], as the students obviously did with only 3% thinking they were below average. In other areas, personality traits have not been found to be highly correlated with overly positive judgments (‘illusory superiority’) [46, 47]. This overconfidence is seen by psychologists as a self-deceiving, probably subconscious, mechanism that cushions a person from experiencing negative feelings [45]. This is probably a normal defence mechanism in a novel situation for many. Overconfidence has been found to be inversely related to diagnostic accuracy to a greater degree in medical practitioners than medical students [18]. However, in this study population, it would be difficult to investigate the association between self-rating and other factors, as there are not enough cases to compare over-rating to normal-rating or under-rating with participants’ judgements clustering around normal and above. A comparison with chiropractic practitioners may clarify if this is the same as for chiropractic students.

### Methodological considerations

Our questionnaire was developed to meet the needs of our objectives, it was pre-tested and refined. Two of the sets of questions (neck and low back pain questionnaires) have been previously used in research [9, 34]; both having been previously tested and refined. In addition, we used a validated questionnaire to measure IU [37].

The response rate was relatively good for one chiropractic program but not as strong for the other. Since the study was anonymous, we could not compare responders with non-responders. However, the profiles for IU were similar in the two programs. We therefore assume that the second smaller sample was not more biased in any particular direction than the larger sample.

The students’ belief in the need for a technique system and their self-rated ability levels did not stratify on IU. It is possible that other psychological profiles would have been better suited to search for explanatory factors associated with different clinical practice styles. Different results may or may not have been found if other measures of chiropractic practitioners’ profiles had been used or the study had been of practising chiropractors. Further, a very high proportion of favourable responses were obtained on the question that asked if students would adopt a prescriptive technique system. It is possible that this question was not as discerning as we intended. This single question was chosen after feedback from chiropractic academics, supervising clinicians and students. However, what may be required is a number of questions, as suggested by more recent research [44]; questions which more fully capture the unknown of clinical practice and thus improves the ability to discern the reasons for wanting to adopt such prescriptive technique systems.

It is also possible that our transformation of the outcome variables could have been performed differently and may have produced different results. We did not analyse the data by year of program, which could have revealed differing levels of knowledge across the year of program of chiropractic students and altered the results. However, this would have been unsuitable as the sample size would have become too small for reliable results when testing the various subgroups.

## Conclusions and recommendations

This study suggests that IU is associated with chiropractic students’ neck pain clinical decisions. If these decisions are unsuitable, this could have implications for health economics because it would result in unnecessary consumption of health resources. It could also, as shown in this study, have deleterious effects on patient safety due to delayed referral. However, IU does not impact all aspects of clinical practice.

The implications for teaching are that students with a high IU appear to make more mistakes on patients with neck pain than with low back pain, which indicates a need for educators to pay special attention to the cervical spine both in relation to indications and contraindications for treatment.

The implications for research are that future studies should refine our understanding of the impact of IU for the case management of low back pain. Also, to learn even more on this topic, it would be useful to test the effect of other psychological profiles and similar work would be relevant in the qualified chiropractor population.

## Additional files

Additional file 1: Anonymous Questionnaire for Chiropractic Students’ Survey. (DOCX 598 kb)

Additional file 2: Explanation of 'correct' and 'incorrect' designation for the neck and low back case scenarios. (DOCX 18 kb)

## Abbreviations

IU: Intolerance of uncertainty
MQ: Macquarie University
MU: Murdoch University
SD: Standard Deviation
