# Supplementary material for: The relationship between intolerance of uncertainty in chiropractic students and their treatment intervention choices
Source: Chiropr Man Therap. 2017 Jul 19;25:20. doi: 10.1186/s12998-017-0150-2 (PMC5518163; doi:10.1186/s12998-017-0150-2)
Supplement: Supplementary file 1 — Anonymous Questionnaire for Chiropractic Students’ Survey. (DOCX 598 kb) [file 12998_2017_150_MOESM1_ESM.docx]

**Additional file 1**

**Anonymous Questionnaire for Chiropractic Students’ Survey**

**Participant consent**

I have read the information letter and I agree to participate in this survey. Your answers are anonymous.

The survey should take about 10 – 15 minutes to complete.

Thank you very much for your assistance.

Stanley Innes

Lecturer Chiropractic / Health Professions

Murdoch University

**Please answer the following questions**

Sex: Male  Female

Year of Program: Yr 1  Yr 2  Yr 3  Yr 4  Yr 5

No or rarely

*Sometimes*

*Quite often*

*Or often*

| 1. **In your practice will you give advice on** |  |  |  |
| --- | --- | --- | --- |
| prevention of stress |  |  |  |
| prevention of cardiovascular disease |  |  |  |
| prevention of diabetes |  |  |  |
| prevention of musculoskeletal problems |  |  |  |
| wellness in general |  |  |  |

*Probably not*

*Definitely not*

*Yes, definitely*

*Don’t know*

*Yes, probably*

| 1. **In your opinion, can chiropractic spinal adjustments** | | | | | |
| --- | --- | --- | --- | --- | --- |
| prevent disease in general? |  |  |  |  |  |
| prevent chronic back pain? |  |  |  |  |  |
| help the immune system? |  |  |  |  |  |
| make it easier to give birth? |  |  |  |  |  |
| improve the health of infants? |  |  |  |  |  |
| help the body function at 100% of its capacity? |  |  |  |  |  |
| prevent degeneration of the spine? |  |  |  |  |  |

1. **When you have graduated would you like to use one or several specific chiropractic technique evaluation systems which tell(s) you what the problem is. For example SOT, Gonstead, Applied Kinesiology or Functional Neurology.**

Yes □ Yes, probably not □ Don’t know □ No, probably □ No □

|  |  |  |  |  |
| --- | --- | --- | --- | --- |

The following questions are about a specific chiropractic technique known as Functional Neurology (FN).

1. **What is your level of knowledge of Functional Neurology (FN)? Select the single best option from the following:**

□ I don’t know anything about FN

□ I have only heard the name FN mentioned without further explanation,

□ I have heard about it discussed by chiropractors and/or students

□ I have seen a chiropractor use it and/or I know someone who has been treated with it

□ I know more than that because I have read about it & /or been to one or several seminar (s)

□ I have been treated with FN myself

□ I have extensive knowledge of FN. Please explain______________________

□ Other: please explain____________________________________________

1. **Would you like to learn (more) how to use Functional Neurology (FN)?**

Yes □ Unsure □ No □

1. **Do you think this technique should be taught in chiropractic programmes?**

Yes mandatory □ As an elective only □ Don’t know □ Probably not□ Definitely not □

1. **Do you think that this technique holds great promise for chiropractic?**

Yes definitely □ Yes probably □ Don’t know □ Probably not □ Definitely not□

1. **CASE SCENARIO A:** The following question is seeking your response to a specific case history. Please read the case history and circle what you think is the most appropriate option from A through to F in each case, numbered from 1 to 5.

A 28-year old man, tennis player by profession, consults you for a right-sided intense neck pain without any radiating pain. You note an antalgic position of the head, no other musculoskeletal signs (no torticollis), no other health problems in particular, normal x-rays for his age, and there are no red flags.

In each of the cases described below, what would you do?

**Answer Options:**

1. I would treat the patient on my own.
2. I would treat the patient with the assistance of some paramedics & / or physiotherapist.
3. I would treat the patient with the assistance of a general practitioner.
4. I would treat the patient whilst asking the opinion of a specialist such as a neurologist / rheumatologist.
5. I would not treat the patient but refer him out.
6. Other, please explain at the bottom of the page.

1. “Physical examination: very tense cervical musculature, no neuro-vascular problems, right C5-6 painful on palpation, pain 7/10 on a visual analogue scale”. **Answer A B C D E F other ____________________**


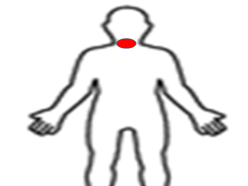


2. “Five days later the patient comes back to you: Same clinical signs but the pain now radiates into the right shoulder”. **Answer A B C D E F other ______________________**


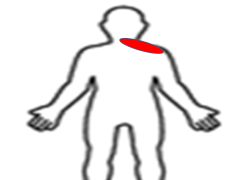


3. “Four days later: An MRI reveals a postero-lateral disc herniation at C5-6 which affects the C6 nerve root. There is now a neurological sign: C6 reflex diminished (1+), normal myotomes and dermatomes.”

**Answer A B C D E F other___________________________**


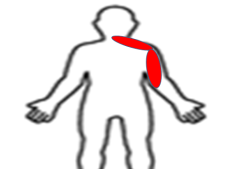


4. “Another 4 days later: The neck pain is gone. The neurological signs are obvious: C6 reflex absent (graded as 0), the C6 myotome diminished (graded as 2), C6 dermatome disturbed (reduced sensitivity).”

**Answer A B C D E F other ___________________________**


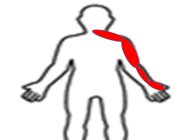


5. The pain drawing is the same and the accompanying text said: “Ten days later: The symptoms and signs are the same as last time but in addition the following is noted: the right leg shows hyperreflexia (graded as 3+), a positive sign of Babinski on the right and slight hypoaesthesia of the right leg.”

**Answer A B C D E F other____________________________**


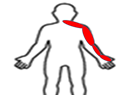


1. **CASE SCENARIO B**

*Please read the following cases and for each case, give the answer that you consider fits best with the decision you would make in a clinical setting.*

*We have selected an imaginary patient, as described in the box below. Then, different scenarios for this patient are outlined, and you are asked to select ONE of several clinical solutions (A,B,C, etc) as listed in bold letters below.*

*You can choose between the following possibilities for each of the cases presented below:*

1. **I would refer the patient to another health care practitioner for a second opinion.**
2. **I would advise the patient to seek additional treatment whilst following the patient.**
3. **I would tell the patient that the treatment is completed but that he is welcome to make a new appointment if the problem returns.**
4. **I would not consider the treatment to be fully completed and would try a few more treatments and perhaps change my treatment strategy, until I am sure that I cannot do any more.**
5. **I would follow this patient for a while, attempting to prolong the time period between visits until either the patient is asymptomatic or until we have found a suitable time lapse between check-ups to keep the patient symptom-free.**
6. **I would recommend that the patient continue with regular visits, as long as clinical findings indicate treatment (eg spinal dysfunction/subluxation) even if the patient is symptom-free.**
7. **None of the above. (Please explain at the bottom of this page in legible handwriting).**

These are the basic facts for our hypothetical patient.

A 40-year old man who consults with you for low back pain with no additional spinal or musculoskeletal problems, and with no other health problems.

His x-rays are normal for his age. There are no “red flags”.

The case above could proceed in the **following 9 ways** described on the next page.

1. **Please circle the number that best corresponds to how much you agree with each item.**

|  | Not at all characteristic of me | A little characteristic of me | Somewhat characteristic of me | Very characteristic of me | Entirely characteristic of me |
| --- | --- | --- | --- | --- | --- |
| 1. Unforeseen events upset me greatly. | 1 | 2 | 3 | 4 | 5 |
| 2. It frustrates me not having all the information I need. | 1 | 2 | 3 | 4 | 5 |
| 3. Uncertainty keeps me from living a full life. | 1 | 2 | 3 | 4 | 5 |
| 4. One should always look ahead so as to avoid surprises. | 1 | 2 | 3 | 4 | 5 |
| 5. A small unforeseen event can spoil everything, even with the best of planning. | 1 | 2 | 3 | 4 | 5 |
| 6. When it’s time to act, uncertainty paralyses me. | 1 | 2 | 3 | 4 | 5 |
| 7. When I am uncertain I can’t function very well. | 1 | 2 | 3 | 4 | 5 |
| 8. I always want to know what the future has in store for me. | 1 | 2 | 3 | 4 | 5 |
| 9. I can’t stand being taken by surprise. | 1 | 2 | 3 | 4 | 5 |
| 10. The smallest doubt can stop me from acting. | 1 | 2 | 3 | 4 | 5 |
| 11. I should be able to organize everything in advance. | 1 | 2 | 3 | 4 | 5 |
| 12. I must get away from all uncertain situations. | 1 | 2 | 3 | 4 | 5 |

**AND FINALLY . . .**

1. **How do you think you will rate as a chiropractor compared to other chiropractors in your class?**

Below Average□ A bit below average □ Average□ A bit above average □

Above average □ Don’t know□

**This completes the survey and please check you have answered all the questions.**
