# Supplementary material for: The relationship between intolerance of uncertainty in chiropractic students and their treatment intervention choices
Source: Chiropr Man Therap. 2017 Jul 19;25:20. doi: 10.1186/s12998-017-0150-2 (PMC5518163; doi:10.1186/s12998-017-0150-2)
Supplement: Supplementary file 2 — Explanation of 'correct' and 'incorrect' designation for the neck and low back case scenarios. (DOCX 18 kb) [file 12998_2017_150_MOESM2_ESM.docx]

**Additional file 2**

**Neck Pain Case**

Below is a description of the five specific management strategies for the neck pain case scenario receiving chiropractic care from which the participants in the survey could select one for each of the five scenarios. The history was for a 28-year old man, tennis player by profession, presenting with right-sided intense neck pain.

The five options were :

1. I would treat the patient on my own.
2. I would treat the patient with the assistance of some paramedics & / or physiotherapist.
3. I would treat the patient with the assistance of a general practitioner.
4. I would treat the patient whilst asking the opinion of a specialist such as a neurologist / rheumatologist.
5. I would not treat the patient but refer him out.
6. Other, please explain at the bottom of the page.

A description of the five scenarios, together with the clinical reasoning (cases 1-5) of the research team for the preferred management strategy for each scenario is provided below.

Case 1. “*Physical examination: very tense cervical musculature, no neuro-vascular problems, right C5-6 painful on palpation, pain 7/10 on a visual analogue scale*”.

According to the research team, this case indicates a person without a background of persistent or recurrent neck pain. It is uncomplicated and with no poor psychological profile which is indicative of a good prognosis. The team would have selected strategy A (‘I would treat the patient on my own’). As such, referral or co-management was unwarranted and deemed to be an unnecessary strategy and an incorrect response.

Case 2. “*Five days later the patient comes back to you: Same clinical signs but the pain now radiates into the right shoulder*”.

Although the pain had spread to the shoulder, there was no other additional neurological progression or other signs or symptoms of deterioration. This case was thought by the team as best being thought of as uncomplicated non-specific neck pain. As such, referral or co-management was unwarranted and deemed to be an unnecessary strategy. Thus the strategy the team would have selected would have been A (‘I would treat the patient on my own’).

Case 3. “*Four days later: An MRI reveals a postero-lateral disc herniation at C5-6 which affects the C6 nerve root. There is now a neurological sign: C6 reflex diminished (1+), normal myotomes and dermatome*s.”

The thoughts of the research team were that this patient had not improved as should be expected. Conservative care had not generated any positive response and hard neurological signs as well as progressively deteriorating symptoms were now evident. The team felt that a change of strategy, referral or second opinion would be a reasonable clinical decision. Thus, they would have selected B, C, D, or E. However, the DTR C6 reflex could be potentially regarded as equivocal, thus the response of A was thought not to be unreasonable. This created the dilemma of all five responses as possibly being correct. Subsequently, the scenario was removed from the analysis.

Case 4. “*Another 4 days later: The neck pain is gone. The neurological signs are obvious: C6 reflex absent (graded as 0), the C6 myotome diminished (graded as 2), C6 dermatome disturbed (reduced sensitivity)*.”

This patient is not improving at a level that should be expected despite the number of visits. The patient is resistant to the type of treatment that has been provided so far. There has been evidence of progressive neurological deterioration and symptomatology. The team determined that more treatment was not warranted. Further, the patient should be referred out and that this referral should have the capacity for advanced imaging as well as more aggressive interventions. Thus the team determined the strategy should have been E (‘I would not treat the patient but refer him out”). Any other choice was deemed to be incorrect.

Case 5. *The pain drawing is the same and the accompanying text said: “Ten days later: The symptoms and signs are the same as last time but in addition the following is noted: the right leg shows hyperreflexia (graded as 3+), a positive sign of Babinski on the right and slight hypoaesthesia of the right leg*.”

There has now been a considerable time lapse with significant and serious signs and symptoms. The team determined that this was now a serious scenario well beyond the scope of conservative care and would require immediate referral. The team would have selected E as the appropriate strategy (‘I would not treat the patient but refer him out”). Any other choice was deemed to be incorrect.

This research team then calculated the number of correct responses for each respondent. A maximum score of 4 and a minimum score of 0 was possible.

**Low Back Pain Case**

A description of nine scenarios (cases 1–9), together with the clinical reasoning of the research team, and a description of their preferred management strategy for each scenario (not included in the questionnaire) is provided below. This is adapted and discussed in relation to Axen et al., Additional File 3 [9].

**Inappropriate “Referral” Strategy - Scenarios 1, 2, and 4.**

*Case 1. An acute attack of low back pain of 2 days’ duration and no previous history of low back pain. The pain is completely gone after two visits. The patient seems to be an uncomplicated person and capable to look after himself and his back.*

According to the Axen et al. 2008 research team, this case indicates a person without a background of persistent or recurrent low back pain, with a quick recovery and a psychological profile that indicates a good prognosis. The Axen et al. team would have selected strategy C (“I would tell the patient that the treatment is completed but that he is welcome to make a new appointment if the problem returns”). Our research team selected the response A as being an unnecessary and inappropriate referral in an uncomplicated simple case of low back pain and was indicative of high levels of IU. Respondents who selected A were given a score of 1.

*Case 2. An acute attack of low back pain of 2 days’ duration and no previous history of low back pain. The pain is completely gone after two visits. The patient is very worried that the pain will come back again. The patient asks if he could come back regularly to make sure this will not happen.*

The thoughts of the Axen et al. research team were that, ideally, this patient should be dismissed, similarly to the case above (strategy C). However, the psychological profile of this patient needs to be taken into account and he should be provided with a sense of security whilst guided by the chiropractor and gradually weaned off to prevent dependency upon chiropractic treatment. The Axen et al. team therefore selected strategy E, with the intent of using a couple more visits to improve the patient’s self-confidence. Our research team selected the response A as being an unnecessary and inappropriate referral in an uncomplicated simple case of low back pain, despite the anxiety of the patient and was also thought to indicate higher levels of IU. Respondents who selected A were again given a score of 1.

*Case 3. An acute attack of low back pain of 2 days’ duration and no previous history of low back pain. The pain is about 20% better after 6 visits.*

This patient was not improving at a level and rate that should be expected. Because the basic case states that there are no red flags, the Axen et al. team decided that this case should be reconsidered and a few more attempts made. The strategy that best suited this scenario was D. We did not use this strategy in our analysis as the response did not allow for a clear delineation of an unsuitable referral or practice type.

*Case 4. An acute attack of low back pain of 1 week’s duration. The patient has had several similar attacks over the past 12 months. The pain is completely gone after 2 weeks of treatment.*

This is a recurrent problem according to the past history. If the patient considers that the chiropractic treatment shortened the duration of the typical attack, he should simply return as soon as a new problem is felt to commence. Unfortunately, many patients will fail to do so, thinking that the treatment did not help when it starts up again. The Axen et al. team decided that it might therefore be advantageous to keep an eye on the patient for a while with the intent of finding out if each event of low back pain can be quickly and efficiently treated at a ‘cost-effect’ time interval (strategy E) or if it is possible to prevent further events (strategy F).

Our research team selected the response A as being an unnecessary and inappropriate referral in this recurrent but uncomplicated case of low back pain. This response was thought to be indicative of higher levels of IU. Respondents who selected A were again given a score of 1.

Consequently, participants’ responses of A (referral), that were thought to reflect higher levels of IU, were scored as 1. These were summed for Cases 1, 2, and 4 to produce a score for each respondent that could range between 0 and 3.

**Inappropriate “Management” Strategy - Scenarios 6, 7, 8 and 9.**

*Case 5. An acute attack of low back pain of 1 week’s duration. The patient has had several similar attacks over the past 12 months, but the pain pattern has varied over the treatment period and now, after six visits, the pain is 20% better.*

This patient is not improving at a level that should be expected despite the large number of visits, indicating that he may be resistant to the type of treatment that has been provided so far. Axen et al. decided that a change of strategy would be required (strategy D) or if the patient is referred out, it would be relevant to keep in touch to be able to be of support in the continued process (strategy B). This case was not used in our study.

*Case 6. The patient has had low back pain intermittently over the past year. After the 2^nd^ visit, the pain was 50% better but today, after six visits there has been no further change.*

The study by Axen et al. interpreted this patient as having reached his optimal stage with the present type of treatment and the therapy should, at this stage, either be reconsidered “in-house” or by someone else, indicating strategy A or D.

For the purposes of our study, the research team deemed that the selection of the response based on clinical findings guided maintenance (subluxation / spinal dysfunction) care (Option F) was indicative of an unsuitable practice profile. This decision was thought to represent a need to have a more rigid and prescriptive technique structure which would inform the practitioner what to do. This was hypothesised to be indicative of higher levels of IU. This rationale that formed the basis of our hypothesis and selection for option F was also applied to Cases 7, 8 and 9.

*Case 7. The patient has had low back pain intermittently over the past year. After 6 visits, the pain was 80% better, but after a further two treatments the last month, the problem has gradually got a bit worse.*

The Axen et al. team used the following reasoning: The improvement seen, to date, may have been independent of the treatment and merely an expression of the typical intermittent pain pattern, or the treatment did have an effect but there is something that re-aggravated the condition. The Axen et al. team therefore reconsidered the case (strategy D) or sent the patient out for an adjunctive approach, such as training, whilst keeping in touch (strategy B).

As previously explained in Case 6, option F was selected by our research team as the incorrect response. This rationale and selection was used again in Cases 8 and 9.

*Case 8. The patient has had low back pain intermittently over the past year. After the 2^nd^ visit, the pain was 20% better, but today, after 6 visits and over the past month, the patient has gradually got worse.*

This patient has not really exhibited a positive response to the treatment and is, in fact, getting worse. That the patient is gradually worsening is not a normal pattern. Despite the fact that there are no (obvious) red flags, the team would refer the patient for a second opinion (strategy A), because some underlying explanatory condition could have been missed. Again option F was deemed to be inappropriate by our research team.

*Case 9. The patient has had low back pain intermittently over the past year. After 6 visits the pain is 20% better. The symptoms come and go for no apparent reason. The patient appears tired and moody.*

This patient has not improved at all and there is no obvious (biomechanical) explanation for the intermittent pattern. There are no red flags but there is a need to consider if there might not be an underlying depression or some other disease, afterall. The team would not hesitate to refer out for a second opinion (strategy A).

Scores for cases 6,7,8 and 9 were summed. This produced a possible score for each respondent which could range between 0 and 4.
